# A new chemoinformatics approach with improved strategies for effective predictions of potential drugs

**DOI:** 10.1186/s13321-018-0303-x

**Published:** 2018-10-11

**Authors:** Ming Hao, Stephen H. Bryant, Yanli Wang

**Affiliations:** National Center for Biotechnology Information, National Library of Medicine, National Institutes of Health, Bethesda, MD 20894 USA

## Abstract

**Background:**

Fast and accurate identification of potential drug candidates against therapeutic targets (i.e., drug–target interactions, DTIs) is a fundamental step in the early drug discovery process. However, experimental determination of DTIs is time-consuming and costly, especially for testing the associations between the entire chemical and genomic spaces. Therefore, computationally efficient algorithms with accurate predictions are required to achieve such a challenging task. In this work, we design a new chemoinformatics approach derived from neighbor-based collaborative filtering (NBCF) to infer potential drug candidates for targets of interest. One of the fundamental steps of NBCF in the application of DTI predictions is to accurately measure the similarity between drugs solely based on the DTI profiles of known knowledge. However, commonly used similarity calculation methods such as COSINE may be noise-prone due to the extremely sparse property of the DTI bipartite network, which decreases the model performance of NBCF. We herein propose three strategies to remedy such a dilemma, which include: (1) adopting a positive pointwise mutual information (PPMI)-based similarity metric, which is noise-immune to some extent; (2) performing low-rank approximation of the original prediction scores; (3) incorporating auxiliary (complementary) information to produce the final predictions.

**Results:**

We test the proposed methods in three benchmark datasets and the results indicate that our strategies are helpful to improve the NBCF performance for DTI predictions. Comparing to the prior algorithm, our methods exhibit better results assessed by a recall-based evaluation metric.

**Conclusions:**

A new chemoinformatics approach with improved strategies was successfully developed to predict potential DTIs. Among them, the model based on the sparsity resistant PPMI similarity metric exhibits the best performance, which may be helpful to researchers for identifying potential drugs against therapeutic targets of interest, and can also be applied to related research such as identifying candidate disease genes.

## Background

A key component in the drug discovery process is to accurately identify the drug–target interactions (DTIs). Traditionally, experimental determination of DTIs is both costly and time consuming. In addition, to fully explore the growing chemical and genomic (for drug targets) spaces being discovered, it becomes impractical to experimentally validate all possible combinations of drug–target pairs. Thus, effective computational algorithms used for predicting potential DTIs are increasingly in demand. Typically, docking simulation is often used to probe the interactions between a series of small molecules and a target under study [1] at a molecular level. However, docking methods require accurate three-dimensional structures of target proteins, making such studies challenging for membrane proteins due to the challenge of protein crystallization. Quantitative structure–activity relationship (QSAR) is another method to depict possible DTIs. However, QSAR typically requires molecular structures with similar scaffolds [2–4] for stronger performance. Nowadays, the technology advancement of next-generation sequencing (NGS) and small molecule high-throughput screening (HTS) is accelerating the identification of potential therapeutic targets and drug compounds, which presents great challenges as well as opportunities for chemogenomic research to explore both chemical and genomic spaces simultaneously. In line with this, Yamanishi et al. [5] proposed a bipartite graph learning method correlating the chemical/genomic spaces with the interaction space (i.e., pharmacological space) for predicting potential DTIs, which was followed by several algorithms with improved performance. For example, Bleakley et al. [6] proposed a novel supervised inference method to predict unknown DTIs by using several bipartite local models (BLM). Specifically, BLM transformed the edge prediction problem into the binary classification problem of points with labels. van Larrhoven et al. [7] used a regularized least squares algorithm combined with the Gaussian interaction profile (GIP) kernel calculated only from the topological information of the drug–target network for inferring DTIs. Mei et al. [8] introduced a neighbor-based interaction-profile inferring method and integrated it into BLM, enabling the model for predicting new drugs/targets. Hao et al. [9] employed a nonlinear kernel diffusion (KF) technique to infer DTIs. Liu et al. [10] proposed a neighborhood regularized logistic matrix factorization (NRLMF) algorithm to partly overcome the imbalanced problem in the DTI prediction process. Later on, Hao et al. [11] designed a dual-network integrated logistic matrix factorization (DNILMF) technique by incorporating an idea for modeling social ensemble into the DTI prediction model. Recently, Olayan et al. [12] proposed a method (called DDR), which is based on heterogeneous graph including known DTI network and multiple similarities from both targets and drugs, to predict unknown DTIs by using Random Forest as a classifier. By adding a heuristic selection of similarity matrices and nonlinear KF technology, DDR outperformed other state-of-the-art priors [12]. Additionally, many other DTI prediction algorithms developed previously can be found in the reviews [13–17].

Among popular DTI prediction algorithms, the most reliable and accurate ones are those based on similarities. However, the used similarity information is derived either from protein sequences or from drug structures. Despite of the importance of the DTI graph, little studies considered using its similarity information as the main source when building the model with the exception of previous work [7]. In fact, the DTI bipartite network itself contains extremely important information, which will be beneficial to the model performance. The success of recommender system in e-commence has provided a proof of concept [18–21], which explores the bipartite network solely. Inspired by this technology, we in this work make an effort to apply and extend it for DTI predictions. Herein, we adopt a technology, called neighbor-based collaborative filtering (NBCF), which is one of the most successful technologies in the community of recommendations. For applying NBCF to DTI predictions, a fundamental step is to accurately compute the pairwise drug similarities based on the drug interaction profiles (DIPs) with targets in the bipartite interaction network, rather than based on drug structures as used in the previous studies. In fact, a similar idea has been reported whereas the protein similarities were measured based on their associated ligands but not based on amino acid sequences [22, 23]. With the DIPs-based drug similarities, an intuitive model is built using NBCF by making the following assumption: if drug A and drug B are highly similar (again, as indicated by similarity from DIPs), and if drug A interacts with the current target, then drug B has a high probability of interacting with the same target, though there may be exceptional cases [22, 23]. However, it is well-known that the experimentally validated interaction information is extremely limited compared to the whole drug–target interaction space, which will introduce noise when computing similarity from such a sparse network (sparseness, defined as the number of links divided by the total number of possible target-drug pairs) using the conventional similarity calculation methods. To tackle this challenge, we in this work propose three strategies to remedy the issue, i.e., by designing a new similarity metric to mitigate noise, performing low-rank approximation (LRA) of the original prediction scores, and incorporating the auxiliary information into the model.

It is critical to select an appropriate evaluation method in order to assess the strength of a developed DTI prediction algorithm as well as to identify rooms for further improvement. Instead of adopting the commonly used evaluation metrics [i.e., area under curve (AUC) and area under precision-recall (AUPR) curve], we introduce a recall-based metric, namely mean percentile ranking (MPR), which is under-studied in DTI predictions [17] but routinely used in the recommender system studies [18, 24]. The reason for selecting MPR as the evaluation criteria is that one only knows about the one-class experimentally validated information (i.e., a drug interacts with a target, which is considered as the positive information) but does not know about the negative information (i.e., a drug does not interact with a target) due to the lack of comprehensive experimental data on a drug–target pair. Thus, a recall-based metric is suitable to such a scenario. Finally, we validate our method in three large publicly available datasets and compare the proposed algorithm with the prior art based on MPR. We conclude that the proposed NBCF algorithm with the improved strategies is both effective and computationally efficient for DTI predictions, which outperforms the previously developed algorithm for identifying potential drugs against therapeutic targets under a study.

## Material and experimental methods

### Datasets

Three large benchmark datasets were used to evaluate the current proposed NBCF algorithm for DTI predictions. The first dataset (denoted by DATASET-H) was derived from our previous work [11], which consists of 733 targets and 829 drugs with 3688 known DTI pairs. This dataset was obtained based on the DrugBank database [25] followed by several pre-processing operations including removing duplicated molecules, mapping to unique identifiers and a few other steps as described previously [11]. The second dataset (denoted by DATASET-K) was retrieved from the study of Kuang et al. [26], which includes 809 targets and 786 drugs forming 3681 known DTI interactions. In this dataset, the drugs were approved by FDA, assigned with at least one ATC code, and the drug data were deposited in the KEGG database [27]. The third dataset (denoted by DATASET-Y) includes 664 targets and 445 drugs with 2926 experimentally validated interactions, which was studied by Yamannishi et al. [5]. Specifically, DATASET-Y was retrieved from multiple databases including KEGG BRITE [27], BRENDA [28], SuperTarget [29] and DrugBank [25]. All benchmark datasets used in this work consist of three matrices: (1) drug–target interaction (adjacency) matrix, denoted by $Y \in {\mathbb{R}}^{M \times N}$ with *M* targets and *N* drugs; (2) target sequence similarity matrix, denoted by $S_{T} \in {\mathbb{R}}^{M \times M}$, calculated from target sequences; and (3) drug structural similarity matrix, denoted by $S_{D} \in {\mathbb{R}}^{N \times N}$, computed from drug chemical structures. Matrix *Y* is often filled by binary numbers, where $Y_{ij} = 1$ if target *i* is targeted by drug *j* validated by the previous experiment, and otherwise $Y_{ij} = 0$ (indicating that drug–target interaction information for the specific pair is unknown). Table 1 shows the benchmark datasets as well as corresponding properties used in this work.

**Table 1:** Benchmark datasets and corresponding properties

	DATASET-H	DATASET-K	DATASET-Y
Number of targets	733	809	664
Number of drugs	829	786	445
Number of interactions	3688	3681	2926
Average interaction number of each drug with targets	~ 4	~ 5	~ 7
Average interaction number of each target with drugs	~ 5	~ 5	~ 4
Minimum interaction number of each drug with targets	1	1	1
Maximum interaction number of each drug with targets	48	48	96
Minimum interaction number of each target with drugs	1	1	1
Maximum interaction number of each target with drugs	75	55	61
Sparsity	0.006	0.006	0.010

### Workflow of the proposed algorithm

**Fig. 1:**
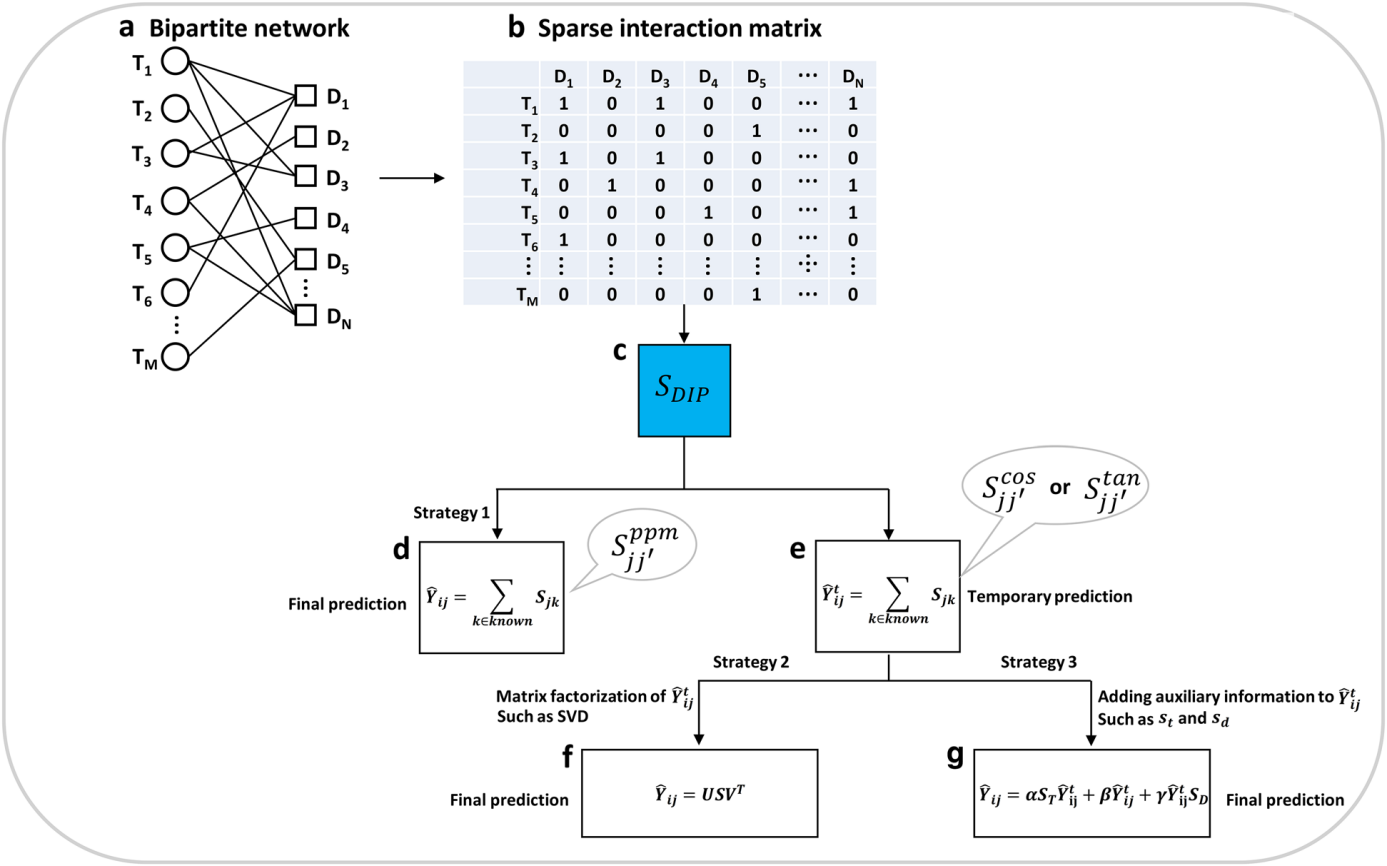
Workflow of the proposed NBCF algorithm with strategies designed for improving DTI predictions

The task of DTI predictions considered in the work is to identify drugs that have larger possibilities of interacting with the targets of interest. Specifically, given a series of targets and drugs, as well as a very small number of known (experimentally determined) interactions, a bipartite network was constructed as shown in Fig. 1a. The bipartite network was converted into an adjacency matrix (also called drug–target interaction matrix), which is very sparse due to the extremely low number of experimentally validated interactions compared to the whole drug–target pair space (shown in Fig. 1b). While “1” is used to indicate a known (positive) interaction, “0” is used to indicate that it is unknown whether the corresponding drug and target interact with each other, because an experiment has not been performed. Based on the sparse interaction matrix, we proposed to use NBCF to infer the potential interactions for those drug–target pairs labelled as 0 s. The development of NBCF was based on a hypothesis that if a query target T_1_ has been reported to interact with drugs of D_1_, D_2_ and D_3_ that are very similar to D_N_, then T_1_ has a large probability for interacting with D_N_. While it is true that chemicals with similar structures do not always exert the same biological properties depending on the similarity degree (e.g., activity cliff) [22, 23, 30], chemical similarity is still a significant principle used when searching for compound candidates for the desired biological activity in drug design and development [31]. Evidently, the key step of NBCF is to accurately assess the pairwise similarity between drugs. Being different from the previous algorithms such as BLM [6] whereas the prior similarity information from drug structures and protein sequences, such as *S*_T_ or *S*_D_, was used as the input (kernel) matrix of support vector machine (SVM) and a conventional binary classification was performed with the fixed regularization parameter *C* of 1, the NBCF technique proposed in the work mainly depends on the similarity information calculated from DIPs in the drug–target interaction matrix, which is denoted by *S*_DIP_ as shown in Fig. 1c. It is well-known that there are multiple methods to calculate similarity from DIPs, two commonly used ones reported here are COSINE and TANIMOTO. For the COSINE similarity, it is defined as follows:

1 $$S_{jj'}^{cos} = \frac{{\mathop \sum \nolimits_{i = 1}^{M} Y_{ij} Y_{ij'} }}{{\sqrt {\mathop \sum \nolimits_{i = 1}^{M} Y_{ij}^{2} } \sqrt {\mathop \sum \nolimits_{i = 1}^{M} Y_{{ij^{\prime}}}^{2} } }} ,$$

where $S_{jj'}^{cos}$ denotes the COSINE similarity between drug *j* and drug *j*’ with the range from − 1 to 1, and *M* is the number of targets. The TANIMOTO similarity (coefficient) is defined as follows:

2 $$S_{{jj^{\prime } }}^{tan} = \frac{{\mathop \sum \nolimits_{i = 1}^{M} Y_{ij} Y_{{ij^{\prime } }} }}{{\mathop \sum \nolimits_{i = 1}^{M} Y_{ij}^{2} + \mathop \sum \nolimits_{i = 1}^{M} Y_{{ij^{\prime } }}^{2} - \mathop \sum \nolimits_{i = 1}^{M} Y_{ij} Y_{{ij^{\prime } }} }} ,$$

where $S_{{jj^{\prime}}}^{tan}$ denotes the TANIMOTO similarity between drug *j* and drug *j*′ with the range from 0 to 1. In addition to the two commonly used similarity calculation methods based on the binary data, we also proposed to use positive pointwise mutual information (PPMI) to measure the similarity between a drug pair. The PPMI approach, which is under-studied in DTI research, has been reported to be a similarity metric, which can mitigate the data sparsity issue to some extent [32]. In the sparse DTI network, the PPMI similarity is defined as follows:

3 $$S_{{jj^{\prime}}}^{ppm} = { \hbox{max} }\left( {log\frac{{P\left( {Y_{.j} ,Y_{{.j^{\prime}}} } \right)}}{{P\left( {Y_{.j} } \right)P\left( {Y_{{.j^{\prime}}} } \right)}},0} \right) ,$$

where the probabilities $P\left( {Y_{.j} ,Y_{{.j^{\prime}}} } \right)$ and $P\left( {Y_{.j} } \right)$ are estimated empirically as follows:

4 $$P\left( {Y_{.j} ,Y_{{.j^{\prime } }} } \right) = \frac{{co\left( {Y_{.j} ,Y_{{.j^{\prime } }} } \right)}}{{\mathop \sum \nolimits_{r,s = 1}^{N} co\left( {Y_{.r} ,Y_{.s} } \right)}} ,$$

5 $$P\left( {Y_{.j} } \right) = \frac{{\mathop \sum \nolimits_{k = 1}^{N} co\left( {Y_{.j} ,Y_{.k} } \right)}}{{\mathop \sum \nolimits_{r,s = 1}^{N} co\left( {Y_{.r} ,Y_{.s} } \right)}} ,$$

where $co\left( {Y_{.j} ,Y_{{.j^{\prime}}} } \right)$ is the number of times that drugs *j* and *j*′ co-occur calculated by summing both co-occurred ones and zeroes in the matrix *Y*, and *N* is the number of drugs. It should be noted that $S_{{jj^{\prime } }}^{ppm}$ is non-negative by replacing negative values to zeroes, and hereby the base 2 logarithm was used in Eq. (3). After yielding three similarity matrices ($S_{jj'}^{cos}$, $S_{{jj^{\prime } }}^{tan}$ and $S_{{jj^{\prime}}}^{ppm}$) calculated from the interaction matrix *Y* solely, the proposed NBCF algorithm was used to calculate the prediction scores (Fig. 1d, e), which is defined as follows:

6 $$\hat{Y}_{ij} = \mathop \sum \limits_{k \in known} S_{jk} ,$$

where $\hat{Y}_{ij}$ denotes the predicted interaction scores between the target *i* of interest and the query drug *j*. $S_{jk}$ denotes the similarity values (i.e., those from either $S_{jj'}^{cos}$, $S_{{jj^{\prime}}}^{tan}$ or $S_{{jj^{\prime}}}^{ppm}$) between the query drug *j* and drugs with known interaction information for the current target *i*. It should be pointed out that, while being simple and intuitive, the proposed algorithm is effective and computationally efficient for DTI predictions due to the model-free property similarly as reported by the previous studies [22, 23]. In fact, a similar algorithm has been successfully applied in the field of recommender systems [19, 21, 33]. However, it should be emphasized that the DTI interaction matrix is extremely sparse, therefore the calculated similarity matrix may include noise, which will decrease the model performance [19]. Thus, we proposed three strategies in the work to overcome such a dilemma. Strategy 1: we designed a similarity calculation algorithm, which is immune to the data sparsity issue to certain degree, with the final generated similarity $S_{{jj^{\prime}}}^{ppm}$. As shown in Fig. 1d, the final prediction scores were obtained directly by using Eq. (6) based on $S_{{jj^{\prime}}}^{ppm}$. Moreover, if the commonly used similarity calculation algorithm is used with the generated matrix (i.e., $S_{jj'}^{cos}$ or $S_{{jj^{\prime}}}^{tan}$), despite that the prediction scores are calculated by using Eq. (6), the scores would be considered as temporary ones as denoted by $\hat{Y}_{ij}^{t}$ as shown in Fig. 1e, due to that $\hat{Y}_{ij}^{t}$ may be sub-optimal because of the noisy similarity information. Thus, we proposed two additional remedy strategies (i.e., Strategy 2 and Strategy 3) to improve the performance on the basis of temporary prediction scores, $\hat{Y}_{ij}^{t}$. Strategy 2: as reported in the community of recommender systems [19], LRA of original prediction scores can help to partially mitigate noise. Thus, we incorporated this technique into the DTI prediction domain. Specially, the singular value decomposition (SVD) algorithm as one of the most popular LRA techniques was adopted to factorize the temporary score matrix, $\hat{Y}_{ij}^{t}$ (Fig. 1f). The final prediction scores were formed according to the following equation:

7 $$\hat{Y}_{ij} = USV^{T} ,$$

where $U \in {\mathbb{R}}^{M \times R}$ is the left singular vector matrix with rank *R* (empirically set to 100), $S \in {\mathbb{R}}^{R \times R}$ is the diagonal matrix, and $V \in {\mathbb{R}}^{N \times R}$ is the right singular vector matrix with $V^{T}$ denoting the transpose of *V*. Strategy 3: while $S_{DIP}$ remains as one of the key components of NBCF derived from the DTI network, the auxiliary similarity information (e.g., $S_{T}$ and $S_{D}$) may be attributed as complementary sources that are beneficial to the model performance. In fact, several previous studies have demonstrated its effectiveness [11, 34]. Therefore, we also explored to include auxiliary information in the NBCF method for the final DTI predictions as defined below (Fig. 1g):

8 $$\hat{Y}_{ij} = \alpha S_{T} \hat{Y}_{ij}^{t} + \beta \hat{Y}_{ij}^{t} + \gamma \hat{Y}_{ij}^{t} S_{D} ,$$

where $\alpha$, $\beta$, and $\gamma$ are the smoothing coefficients (empirically set to 0.025, 0.95 and 0.025, respectively).

### Evaluation method

In this work, we used tenfold cross-validation to evaluate the proposed algorithm. Specifically, we removed randomly a subset of 10% of the links (known interaction pairs) in the drug–target interaction matrix *Y* as the test set and trained models on the remaining links (i.e., 90% of the known interaction pairs). In addition, we ensured each drug has at least one interaction with a target (and vice versa that each target has at least one interaction with a drug as well) similarly to the previous work [35]. We adopted a recall-based evaluation metric, MPR [18, 24], to evaluate the algorithm performance. In detail, for each target *i* in the test set, we generated a ranked list of potential drugs, sorted by a decreasing order according to the final prediction scores for the potential interaction between target *i* and each of the drugs in the dataset. Let rank_ji_ denote the percentile ranking (PR) of target *i* for drug *j*. This way, at rank_ji_ = 0%, drug *j* is predicted as the drug with the highest probability of interacting with target *i*, while at rank_ji_ = 100%, drug *j* is predicted as the drug with the lowest probability of interacting with target *i*. Herein, the definition of MPR is described as follows:

9 $$MPR = \frac{{\mathop \sum \nolimits_{i = 1}^{{N_{T}^{test} }} R_{i} }}{{N_{T}^{test} }},$$

where $N_{T}^{test}$ denotes the number of targets in the test set, and $R_{i}$ is computed as follows:

10 $$R_{i} = \frac{{\mathop \sum \nolimits_{j = 1}^{{N_{D}^{test} }} rank_{ji} }}{{N_{D}^{test} }},$$

where $N_{D}^{test}$ denotes the number of drugs in the test set for the current target *i*. It should be pointed out that the lower MPR is, the more desirable performance the model exhibits, as a lower MPR value indicates the drug–target pair is predicted as interacting with each other with a higher possibility. Evidently, randomly generated lists have an expected MPR of 50% [24]. Using this metric, one can obtain a recommended list of candidate drugs, with top predictions recommended to be given higher priority for experimental validation.

## Results and discussion

### Properties of benchmark datasets

We validate our algorithm using three benchmark datasets (Table 1). (1) DATASET-H: in this dataset which was obtained from our previous work [11], there are 733 unique targets and 829 unique drugs extracted from the DrugBank database following several pre-processing steps. On average, DATASET-H has about 4 known targets for each drug and 5 drugs for each target. Among them, looked from the drug side, the minimum and maximum number of interacted targets are 1 and 48, respectively. From the target end, the minimum and maximum number of interacted drugs are 1 and 75, respectively. The sparsity value (calculated from known interactions divided by the totally possible interaction pairs between drugs and targets; the lower the value, the sparser the dataset is) is 0.006, indicating the dataset is very sparse. (2) DATASET-K: the dataset was retrieved from the publication of Kuang and co-workers [26]. This dataset is similar to DATASET-H, but has more targets than drugs. This dataset also has the sparsity value of 0.006, making the DTI predictions extreme difficult. (3) DATASET-Y: this dataset is a subset of the previous work with the largest number of possible interaction pairs published by Yamanishi and co-workers [5]. Similar to DATASET-K, this dataset also has more targets than drugs. Compared to the first two datasets, the sparsity value of DATASET-Y is relative higher (0.010) indicating it is relatively less (but still very) sparse and has more known interactions within the dataset. In summary, all these three benchmark datasets have a very low sparsity value leaving a larger room for challenging the algorithms for DTI predictions.

### Results of the proposed algorithm

**Fig. 2:**
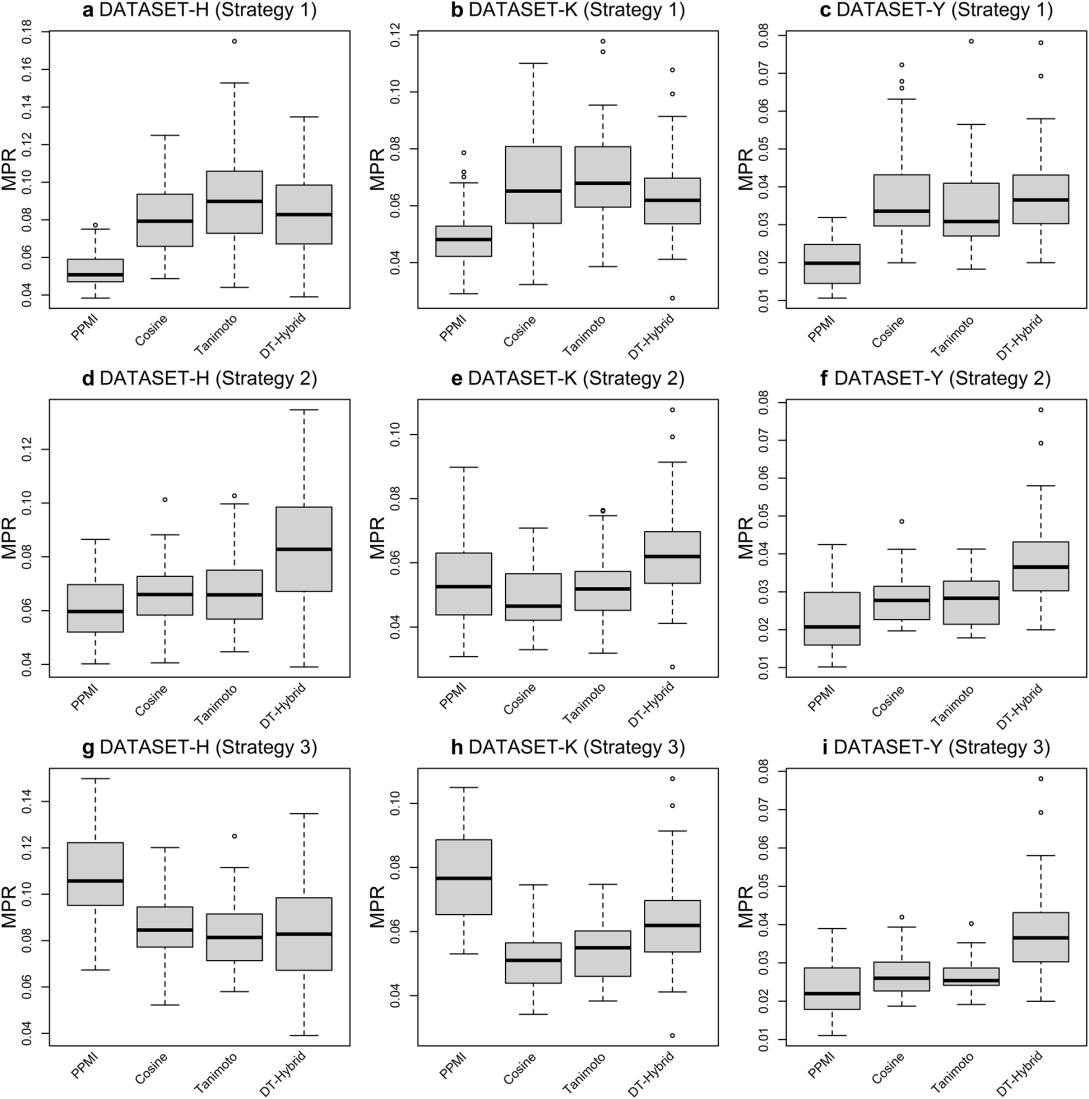
Boxplots of MPR for the proposed NBCF algorithm for three benchmark datasets. **a**–**c** MPR based on Strategy 1; **d**–**f** MPR based on Strategy 2; and **g**–**i** MPR based on Strategy 3

In this section, we evaluate the proposed NBCF algorithm for predicting DTIs using the three extremely sparse benchmark datasets. As shown in Table 2, in Strategy 1 (i.e., results are totally based on $S_{DIP}$ as shown in Fig. 1c–e), results based on PPMI give MPR values as of 0.054, 0.049 and 0.020 for DATASET-H, DATASET-K and DATASET-Y, respectively. COSINE-based MPR values are 0.081, 0.068 and 0.037 for the same datasets, respectively, while TANIMOTO-based MPR values are 0.092, 0.070 and 0.035. From these results, we conclude that the proposed NBCF algorithm has generated promising results which largely outperform the random recommendation accuracy (i.e., 0.5) in terms of MPR [18, 24], and evidently, PPMI-based NCBF significantly outperforms both the COSINE-based and TANIMOTO-based counterparts (*P* < 0.01, *t* test). The observation is not surprising because the similarity information used in the PPMI-based NBCF technique is intentionally designed for overcoming noise from the sparse DTI network, while the NBCF methods based on the COSINE and TANIMOTO similarity metrics exhibit the sub-optimal results due to the noise-prone properties in such commonly used calculation methods. It should be emphasized that while results from the PPMI-based NBCF algorithm are used as the final prediction scores (Fig. 1d), the COSINE and TANIMOT-based ones are considered as the temporary results, denoted by $\hat{Y}_{ij}^{t}$ as shown in Fig. 1e, which can be further improved by our proposed strategies as described in the following. Since the previous study has reported that the LRA operation of original prediction scores can reduce noise to some extent [19], we thus adopt one of the most popular LRA techniques (i.e., SVD) to factorize $\hat{Y}_{ij}^{t}$ and yield the final prediction scores according to Eq. (7), which belongs to Strategy 2. As shown in Table 2, the strategy largely enhances the performance for COSINE-based and TANIMOTO-based NBCF. For example, in DATASET-H, COSINE-based NBCF improved MPR from the original 0.081 to 0.066, and TANIMOTO-based NBCF also produced better MPR results compared to the one in Strategy 1. In DATASET-K and DATASET-Y, the performance of COSINE-based and TANIMOTO-based NBCF in Strategy 2 consistently outperforms those in Strategy 1. These observations indicate that LRA doubtless plays an important role in improving the model performance from COSINE-based and TANIMOTO-based NBCF. However, it is not the case for PPMI-based NBCF, where the performance actually decreases when LRA was applied. This is because similarity based on PPMI used in NBCF has already successfully reduced noise from the sparse DTI network, thus an extra LRA operation by using SVD might be “over killing” and may even affect the results adversely. Moreover, it is interesting to note that in DATASET-K, COSINE-based NBCF in Strategy 2 generated comparable results with PPMI-based one in Strategy 1. Though one of the key ideas of NBCF is to accurately construct the similarity matrix (i.e., $S_{DIP}$ as shown in Fig. 1c) only from the DTI profiles (Fig. 1b), the auxiliary similarity information, such as $S_{T}$ and $S_{D}$ used in this work, may be beneficial to the model performance by incorporating complementary information appropriately, as expected for models from COSINE-based and TANIMOTO-based NBCF especially. Thus, in Strategy 3, we explore such auxiliary information by adding them into the original NBCF model with a similar approach used by the previous studies [11, 34] (Eq. 8). As shown in Table 2, it is evident that both COSINE-based and TANIMOTO-based NBCF models exhibit enhanced performance in all benchmark datasets, with the exception that in DATASET-H, COSINE-based NBCF gave slightly lower performance than that in Strategy 1. However, PPMI-based NBCF in Strategy 3 does not show appreciation for such auxiliary similarity information at all. On the contrary, decreased performance is observed with the incorporation of $S_{T}$ and $S_{D}$, which indicates that the NBCF model on the basis of PPMI can generate the most optimal performance, while extra operations may have an adverse effect on the model. In summary, we conclude that the proposed strategies are undoubtedly playing a central role in improving the DTI prediction performance based on the NBCF model. Among them, PPMI-based NBCF gives the best results in all three benchmark datasets due to the well-designed similarity measurement method, which can effectively tackle the sparsity issue in the DTI network. Moreover, both the LRA operation and incorporation of auxiliary information are helpful to enhance the performance for models that are based on the commonly used similarity metrics such as COSINE and TANIMOTO. Figure 2 shows the corresponding boxplots of all these results.

**Table 2:** Results of MPR for the proposed algorithms based on 5 trials of tenfold cross-validation in the benchmark datasets

Similarity method	DATASET-H	DATASET-K	DATASET-Y
Strategy 1			
PPMI	0.054 ± 0.010	0.049 ± 0.010	0.020 ± 0.006
COSINE	0.081 ± 0.019	0.068 ± 0.019	0.037 ± 0.013
TANIMOTO	0.092 ± 0.026	0.070 ± 0.017	0.035 ± 0.012
Strategy 2			
PPMI	0.061 ± 0.012	0.055 ± 0.014	0.023 ± 0.008
COSINE	0.066 ± 0.013	0.049 ± 0.010	0.029 ± 0.007
TANIMOTO	0.066 ± 0.013	0.052 ± 0.011	0.028 ± 0.007
Strategy 3			
PPMI	0.109 ± 0.020	0.077 ± 0.014	0.023 ± 0.007
COSINE	0.086 ± 0.013	0.051 ± 0.009	0.027 ± 0.006
TANIMOTO	0.083 ± 0.014	0.055 ± 0.010	0.027 ± 0.004
DT-hybrid			
–	0.083 ± 0.023	0.063 ± 0.016	0.037 ± 0.013

### Comparison to counterpart and further consideration

We compared the proposed NBCF algorithm to DT-Hybrid proposed by Alaimo and co-workers [35]. We select DT-Hybrid for comparison because (1) both NBCF and DT-Hybrid are derived from network based recommendation technology [19, 20, 33]; (2) both algorithms adopt a recall-based metrics; and (3) they are both effective and computationally efficient for DTI predictions. For DT-Hybrid, we adopt the default parameters according to the reported values (i.e., lambda set to 0.5 and alpha set to 0.4) [35]. As shown in Table 2, in all three datasets, PPMI-based NBCF in Strategy 1, and both COSINE-based and TANIMOTO-based NBCF models in Strategy 2 generated much better results than those from DT-Hybrid. Similarly, models from COSINE and TANIMOTO in Strategy 3 consistently outperform those from DT-Hybrid. Therefore, all the results indicate that our proposed algorithm with the improved strategies demonstrated stronger prediction ability for inferring potential DTIs. Though the NBCF algorithm combined with similarity from either PPMI, COSINE or TANIMOTO proposed in this work has been successful for DTI predictions, we were interested to explore the effect of other similarity methods on the model performance. Since the GIP kernel was reported to be a useful similarity metric for predicting potential DTIs in the previous work [7, 9, 11], we performed an experiment based on GIP. However, no satisfied results were obtained in all three benchmark datasets in terms of MPR. When we tested another similarity metric called DICE coefficient, results showed similar trend with those based on COSINE and TANIMOTO. Furthermore, we experimented the proposed algorithm with the IC dataset from the previous study [5], and noticed that the PPMI-based model still exhibits the best performance. To further validate the model effectiveness, we also performed fivefold cross-validations, which generated similar results as those from the tenfold cross-validation. It should be noted that the current work mainly focuses on inferring the potential drugs for interesting targets. In fact, the inverse operation (i.e., inferring the potential targets for interesting drugs) is also possible, which will be further explored in the future. Moreover, we plan to improve the current algorithm to make it scalable to larger datasets, and suitable to the new targets (or new drugs) scenarios [8, 10, 11, 36, 37].

## Conclusions

In this work, we propose a straightforward yet effective and computationally efficient algorithm, NBCF, for inferring potential DTIs. For overcoming data sparsity inherently existing in the known DTI network, we designed three strategies to tackle the difficult issue. In Strategy 1, we propose to use a sparsity resistant similarity metric, PPMI, to measure the correlation between drugs from the DTI network solely, which as a result exhibits the best performance in the current work. In Strategies 2 and 3, we apply the low-rank approximation technique and incorporate additional auxiliary similarity into noise-prone models (i.e., COSINE-based NBCF and TANIMOTO-based NBCF) respectively, which have been shown to enhance the prediction accuracy to identify drug candidates for therapeutic targets.
